# Spectral Transition for Dirac Operators with Electrostatic $\delta$-Shell Potentials Supported on the Straight Line

**DOI:** 10.1007/s00020-022-02711-6

**Published:** 2022-08-30

**Authors:** Jussi Behrndt, Markus Holzmann, Matěj Tušek

**Affiliations:** 1 Institut für Angewandte Mathematik, Technische Universität Graz, Steyrergasse 30, 8010 Graz, Austria; 2 Department of Mathematics, Faculty of Nuclear Sciences and Physical Engineering, Czech Technical University in Prague, Trojanova 13, 120 00 Prague, Czech Republic

**Keywords:** Dirac operator, Singular potential, Boundary triple, Spectral transition, Primary 81Q10, Secondary 35Q40

## Abstract

In this note the two dimensional Dirac operator $A_\eta$ with an electrostatic $\delta$-shell interaction of strength $\eta \in {\mathbb {R}}$ supported on a straight line is studied. We observe a spectral transition in the sense that for the critical interaction strengths $\eta =\pm 2$ the continuous spectrum of $A_\eta$ inside the spectral gap of the free Dirac operator $A_0$ collapses abruptly to a single point.

## Introduction

Differential operators that admit a spectral transition are of particular interest in mathematical analysis and its applications. Typically, one expects that the properties of a model described by a differential operator depend continuously on the parameters. However, in some cases it turns out that there is an abrupt change in the spectral properties-in other words, a spectral transition. A well known example in this regard is the Smilansky model [29, 30] (see also [4–6]) or the indefinite Laplacian studied in [13, 20]. A spectral transition was also observed for Dirac operators with singular potentials supported on bounded curves in ${\mathbb {R}}^2$ and surfaces in ${\mathbb {R}}^3$. More precisely, when studying perturbations $A_\eta$ of the free Dirac operator by an electrostatic $\delta$-shell potential of strength $\eta \in {\mathbb {R}}$, it turned out that there is an abrupt change in the spectral properties for $\eta = \pm 2$. While for $\eta \ne \pm 2$-which is referred to as the *non-critical case*-it is shown in the three dimensional situation in [2, 3, 7, 8] that the essential spectrum consists of two unbounded rays and finitely many eigenvalues between these rays, the *critical case* $\eta = \pm 2$ remained initially open. In the critical case it was then proved in [10, 18, 27] that there is a loss of smoothness in the operator domain and that there may be one additional point in the essential spectrum; for general combinations of interaction strengths in the two dimensional setting see [12]. We note that similar effects also appear in the study of Dirac operators on bounded domains with suitable boundary conditions; cf. [11, 17, 18, 21, 26].

In this note we study the spectrum of a Dirac operator in ${\mathbb {R}}^2$ with an electrostatic $\delta$-shell potential of strength $\eta \in {\mathbb {R}}$ supported on the straight line $\Sigma \cong {\mathbb {R}}$ which is formally given by

$$\begin{aligned} A_\eta = -i (\sigma _1 \partial _1 + \sigma _2 \partial _2) + m \sigma _3 + \eta \sigma _0 \delta _\Sigma ; \end{aligned}$$

here and in the following $\sigma _1, \sigma _2, \sigma _3$ are the ${\mathbb {C}}^{2 \times 2}$-valued Pauli spin matrices defined in (1.3), $\sigma _0$ is the $2 \times 2$-identity matrix, and $m \in {\mathbb {R}} \setminus \{ 0 \}$. In order to define this expression rigorously, we denote by ${\mathbb {R}}^2_+$ and ${\mathbb {R}}^2_-$ the upper and lower half plane, respectively, and we use the notation $f_\pm := f\upharpoonright {\mathbb {R}}^2_\pm$ for the restriction of a function *f* defined on ${\mathbb {R}}^2$. Next, let

$$\begin{aligned} H(\sigma , {\mathbb {R}}^2_\pm ) := \big \{ f \in L^2({\mathbb {R}}^2_\pm ; {\mathbb {C}}^2): (\sigma _1 \partial _1 + \sigma _2 \partial _2) f \in L^2({\mathbb {R}}^2_\pm ; {\mathbb {C}}^2) \big \}. \end{aligned}$$

Then there exists a bounded Dirichlet trace map $H(\sigma , {\mathbb {R}}^2_\pm ) \rightarrow H^{-1/2}(\Sigma ; {\mathbb {C}}^2)$, $f_\pm \mapsto f_\pm \vert _\Sigma$ (one shows this in the same way as in [17, Lemma 2.1] and [17, Lemma 2.3] for bounded domains), and this allows us to define for $\eta \in {\mathbb {R}}$ and $m \in {\mathbb {R}} \setminus \{ 0 \}$ the operator

1.1 $$\begin{aligned} \begin{aligned} A_\eta f&:= \big (-i (\sigma _1 \partial _1 + \sigma _2 \partial _2) + m \sigma _3 \big ) f_+ \oplus \big (-i (\sigma _1 \partial _1 + \sigma _2 \partial _2) + m \sigma _3 \big ) f_-, \\ \mathrm {dom}\,A_\eta&:= \bigg \{ f = f_+ \oplus f_- \in H(\sigma , {\mathbb {R}}^2_+) \oplus H(\sigma , {\mathbb {R}}^2_-): \\&\qquad \qquad \qquad i\sigma _2 (f_+|_\Sigma - f_-|_\Sigma ) = \frac{\eta }{2} (f_+|_\Sigma + f_-|_\Sigma ) \bigg \}, \end{aligned} \end{aligned}$$

in $L^2({\mathbb {R}}^2; {\mathbb {C}}^2)$. This operator is the rigorous mathematical definition of a Dirac operator with an electrostatic $\delta$-shell interaction of strength $\eta$ and models, for $m>0$, the propagation of a particle with mass *m* and spin 1/2 under the influence of such a potential; cf. [2, 8, 12, 21]. Observe that for $\eta =0$ the operator in (1.1) coincides with the free Dirac operator

1.2 $$\begin{aligned} A_0 f = -i (\sigma _1 \partial _1 + \sigma _2 \partial _2) f + m \sigma _3 f, \quad \mathrm {dom}\,A_0 = H^1({\mathbb {R}}^2; {\mathbb {C}}^2), \end{aligned}$$

and recall that $A_0$ is self-adjoint in $L^2({\mathbb {R}}^2; {\mathbb {C}}^2)$ with purely (absolutely) continuous spectrum

$$\begin{aligned} \sigma (A_0) = (-\infty , -|m|] \cup [|m|, +\infty ). \end{aligned}$$

The main result of this paper is the following theorem on the self-adjointness and the spectra of the operators $A_\eta$. It turns out that the continuous spectrum grows under the influence of the $\delta$-shell interaction and also half of the gap $(-|m|, |m|)$ is filled when $\eta$ approaches the critical values $\pm 2$ from above or below. In the critical case $\eta =\pm 2$ a spectral transition appears: the continuous spectrum inside $(-|m|, |m|)$ vanishes abruptly and 0 becomes an infinite dimensional eigenvalue.

### Theorem 1.1

The operator $A_\eta$ is self-adjoint in $L^2({\mathbb {R}}^2; {\mathbb {C}}^2)$ and the following holds for the spectrum of $A_\eta$:

(i) If $$\eta < -2$$, then $$\sigma (A_\eta )=\big (-\infty , -|m|\frac{\eta ^2-4}{\eta ^2+4}\big ] \cup [|m|, +\infty )$$.
(ii) If $\eta =- 2$, then $\sigma (A_{\eta }) = (-\infty , -|m|] \cup \{ 0 \} \cup [|m|, +\infty )$.
(iii) If $-2<\eta < 0$, then $\sigma (A_{\eta })=(-\infty , -|m|] \cup \big [|m| \frac{4-\eta ^2}{\eta ^2+4}, +\infty \big )$.
(iv) If $\eta =0$, then $\sigma (A_{0})=(-\infty , -|m|] \cup \big [|m| , +\infty \big )$.
(v) If $$0<\eta < 2$$, then $$\sigma (A_{\eta })=\big (-\infty , -|m|\frac{4-\eta ^2}{\eta ^2+4}\big ] \cup [|m|, +\infty )$$.
(vi) If $\eta = 2$, then $\sigma (A_{\eta }) = (-\infty , -|m|] \cup \{ 0 \} \cup [|m|, +\infty )$.
(vii) If $2<\eta$, then $\sigma (A_{\eta })=(-\infty , -|m|] \cup \big [|m| \frac{\eta ^2-4}{\eta ^2+4}, +\infty \big )$.

For $\eta \not =\pm 2$ the spectrum of $A_\eta$ is purely continuous and for $\eta =\pm 2$ the point 0 is an isolated eigenvalue of $A_\eta$ with infinite multiplicity and the remaining spectrum is purely continuous.

Note that the spectrum of $A_\eta$ is invariant under the transformation $\eta \mapsto -\frac{4}{\eta }$. This symmetry would also follow from the stronger fact that $A_\eta$ and $A_{-4/\eta }$ are unitary equivalent; this can be shown in the same way as in [12, Proposition 4.8 (i) and Proposition 4.15 (i)].

The proof of Theorem 1.1 is based on an efficient abstract technique that was applied in a similar form also in [12, 21]: We use a so-called boundary triple and its Weyl function to reduce the spectral analysis of $A_\eta$ in the gap $(-\vert m\vert ,\vert m\vert )$ of $A_0$ to a certain boundary operator in $L^2(\Sigma ; {\mathbb {C}}^2) \simeq L^2({\mathbb {R}}; {\mathbb {C}}^2)$; cf. [9, 19, 22, 23] for details on boundary triples and Weyl functions in the extension theory of symmetric operators. Since $\Sigma$ is a straight line the spectral properties of this boundary operator can be studied with the help of the Fourier transform. From the limit behaviour of the Weyl function and the boundary operator towards the real line we also conclude that the set $(-\infty , -|m|] \cup [|m|, +\infty )$ consists of purely continuous spectrum of $A_\eta$ for all $\eta \in {\mathbb {R}}$. For $m=0$ this method is also applicable, then it follows for all $\eta \in {\mathbb {R}}$ that $\sigma (A_\eta ) = {\mathbb {R}}$, i.e. there is no spectral transition. We note that the method of direct integrals decomposing $A_\eta$ in its fibers would yield a similar result; cf. [24] for a related problem on Dirac operators with Robin type boundary conditions. In this note we prefer to work with the boundary triple technique, since it allows generalizations for more complicated curves $\Sigma$ in a natural way, which we plan to study in the near future.

### Notations

Let

1.3 $$\begin{aligned} \sigma _1 := \begin{pmatrix} 0 &{} 1 \\ 1 &{} 0 \end{pmatrix}, \qquad \sigma _2 := \begin{pmatrix} 0 &{} -i \\ i &{} 0 \end{pmatrix}, \qquad \sigma _3 := \begin{pmatrix} 1 &{} 0 \\ 0 &{} -1 \end{pmatrix}, \end{aligned}$$

be the Pauli spin matrices and denote by $\sigma _0$ the $2 \times 2$-identity matrix. Note that the Pauli matrices satisfy $\sigma _j \sigma _k + \sigma _k \sigma _j = 2 \delta _{jk} \sigma _0$, $j,k \in \{ 1,2,3\}$. For $x = (x_1,x_2)$ we will often write $\sigma \cdot x = \sigma _1 x_1 + \sigma _2 x_2$ and $\sigma \cdot \nabla = \sigma _1 \partial _1 + \sigma _2 \partial _2$.

## Proof of Theorem 1.1

Let $A_0$ be the free Dirac operator in (1.2) and recall that for $z \in \rho (A_0)$ the resolvent of $A_0$ is given by

$$\begin{aligned} \big [(A_0 - z)^{-1} f\big ](x) = \int _{{\mathbb {R}}^2} G_z(x - y) f(y) d y, \quad f \in L^2({\mathbb {R}}^2; {\mathbb {C}}^2),\, x \in {\mathbb {R}}^2, \end{aligned}$$

where

$$\begin{aligned} G_z(x) = \frac{i\sqrt{m^2 \! -\! z^2}}{2 \pi } K_1 \big (\sqrt{m^2 \! -\! z^2} | x |\big ) \frac{\sigma \cdot x}{ | x | } \!+\! \frac{1}{2 \pi } K_0 \big (\sqrt{m^2 \! -\! z^2} | x |\big )\! \big ( z \sigma _0 + m \sigma _3\big ) \end{aligned}$$

and $K_j$ is the modified Bessel functions of the second kind and order *j*; cf. [1] and [31]. Here and in the following the complex square root is chosen such that it is holomorphic in ${\mathbb {C}} \setminus (-\infty , 0]$ and $\text {Re}\, \sqrt{z} > 0$. An important object in our analysis is the mapping ${\mathcal {C}}_z$ which is defined for $z \in \rho (A_0)$ by

$$\begin{aligned} {\mathcal {C}}_z \varphi := G_z * \varphi , \quad \varphi \in {\mathcal {S}}(\Sigma ; {\mathbb {C}}^2), \end{aligned}$$

where ${\mathcal {S}}(\Sigma ; {\mathbb {C}}^2) \simeq {\mathcal {S}}({\mathbb {R}}; {\mathbb {C}}^2)$ denotes the Schwartz space and the convolution is understood in the sense of distributions. The action of ${\mathcal {C}}_z$ is

$$\begin{aligned} {\mathcal {C}}_z \varphi (x) := \lim _{\varepsilon \searrow 0} \int _{{\mathbb {R}} \setminus (x_1-\varepsilon , x_1+\varepsilon )} G_z(x-y) \varphi (y) d y_1, \quad \!\!x=(x_1,0), y=(y_1,0) \in \Sigma . \end{aligned}$$

In the following we will denote by ${\mathcal {F}}$ the Fourier transform on $\Sigma \simeq {\mathbb {R}}$.

### Lemma 2.1

The map ${\mathcal {F}} {\mathcal {C}}_z {\mathcal {F}}^{-1}$ is the multiplication operator with the matrix valued function

$$\begin{aligned} p\mapsto \begin{pmatrix} \frac{z + m}{2 \sqrt{p^2+m^2-z^2}} &{} \frac{p}{2 \sqrt{p^2+m^2-z^2}} \\ \frac{p}{2 \sqrt{p^2+m^2-z^2}} &{} \frac{z - m}{2 \sqrt{p^2+m^2-z^2}} \end{pmatrix}. \end{aligned}$$

In particular, ${\mathcal {C}}_z$ gives rise to a bounded operator in $H^s(\Sigma ; {\mathbb {C}}^2)$ for any $s \in {\mathbb {R}}$.

### Proof

Let $\phi _z(x_1) := \frac{1}{2 \pi } K_0(\sqrt{m^2-z^2}|x_1|)$, $x_1 \in {\mathbb {R}} \setminus \{ 0 \}$. Since the Fourier transform takes $K_0(\kappa |x|)$ to $\sqrt{\pi /2}(p^2+\kappa ^2)^{-1/2}$ we obtain

$$\begin{aligned} {\mathcal {F}} (\phi _z * \varphi )(p) = \frac{1}{2 \sqrt{p^2+m^2-z^2} } {\mathcal {F}} \varphi (p),\quad \varphi \in {\mathcal {S}}({\mathbb {R}}). \end{aligned}$$

Now observe that

$$\begin{aligned} G_z(x) = \left( -i \sigma _1 \frac{d}{dx_1} + m \sigma _3 + z \sigma _0 \right) \phi _z(x_1),\quad x=(x_1,0) \in \Sigma , \end{aligned}$$

and hence the calculation rules for the Fourier transform of distributions [28, Chapter IX] lead to

$$\begin{aligned} \begin{aligned} {\mathcal {F}} {\mathcal {C}}_z {\mathcal {F}}^{-1} \varphi (p)&= \left( \sigma _1 p + m \sigma _3 + z \sigma _0 \right) \frac{1}{2 \sqrt{p^2+m^2-z^2}} \varphi (p) \\&= \begin{pmatrix} \frac{z + m}{2 \sqrt{p^2+m^2-z^2}} &{} \frac{p}{2 \sqrt{p^2+m^2-z^2}} \\ \frac{p}{2 \sqrt{p^2+m^2-z^2}} &{} \frac{z - m}{2 \sqrt{p^2+m^2-z^2}} \end{pmatrix} \varphi (p) \end{aligned} \end{aligned}$$

for $\varphi \in {\mathcal {S}}(\Sigma ; {\mathbb {R}}^2)$, which yields the claimed result about the representation of ${\mathcal {F}} {\mathcal {C}}_z {\mathcal {F}}^{-1}$. Finally, taking the definition of the norm in $H^s(\Sigma ; {\mathbb {C}}^2) \simeq H^s({\mathbb {R}}; {\mathbb {C}}^2)$ with the help of the Fourier transform into account, one sees that ${\mathcal {C}}_z$ gives rise to a bounded operator in $H^s(\Sigma ; {\mathbb {C}}^2)$ for all $s \in {\mathbb {R}}$. $\square$

In the following we shall make use of the closed symmetric restriction

2.1 $$\begin{aligned} S f = (-i \sigma \cdot \nabla + m \sigma _3) f, \quad \mathrm {dom}\,S = H^1_0({\mathbb {R}}^2 \setminus \Sigma ; {\mathbb {C}}^2), \end{aligned}$$

of the free Dirac operator $A_0$, and its adjoint

$$\begin{aligned} \begin{aligned} S^* f&= (-i \sigma \cdot \nabla + m \sigma _3) f_+ \oplus (-i \sigma \cdot \nabla + m \sigma _3) f_-, \\ \mathrm {dom}\,S^*&= H(\sigma , {\mathbb {R}}^2_+) \oplus H(\sigma , {\mathbb {R}}^2_-); \end{aligned} \end{aligned}$$

the above mentioned properties of *S* and $S^*$ can be shown in the same way as in [10, Proposition 3.1], where similar operators in ${\mathbb {R}}^3$ with compact surfaces $\Sigma$ have been studied.

Fix some $\zeta \in \rho (A_0)$. Define the maps $\Gamma _0, \Gamma _1: \mathrm {dom}\,S^* \rightarrow L^2(\Sigma ; {\mathbb {C}}^2)$ by

2.2 $$\begin{aligned} \begin{aligned} \Gamma _0 f&:= -i \Lambda ^{-1} \sigma _2 \big (f_+\vert _\Sigma - f_-\vert _\Sigma \big ), \\ \Gamma _1 f&:= \frac{1}{2} \Lambda \big ( (f_+\vert _\Sigma + f_-\vert _\Sigma ) - ({\mathcal {C}}_\zeta + {\mathcal {C}}_{{\overline{\zeta }}}) \Lambda \Gamma _0 f \big ), \quad f = f_+ \oplus f_- \in \mathrm {dom}\,S^*, \end{aligned} \end{aligned}$$

where $\Lambda =(-\Delta +1)^\frac{1}{4}={\mathcal {F}}^{-1}(p^2+1)^\frac{1}{4} {\mathcal {F}}$ is viewed as a bijective operator from $H^s(\Sigma )$ onto $H^{s-1/2}(\Sigma )$ for $s=1/2$ or $s=0$, or as a self-adjoint unbounded operator in $L^2(\Sigma )$ defined on $H^{1/2}(\Sigma )$. It follows in the same way as in [12, Proposition 3.5] or [21, Proposition 6.1] that $\{ L^2(\Sigma ; {\mathbb {C}}^2), \Gamma _0, \Gamma _1 \}$ is a boundary triple for $S^*$ with $A_0 = S^* \upharpoonright \ker \Gamma _0$ and corresponding Weyl function

$$\begin{aligned} \rho (A_0) \ni z \mapsto M(z) = \Lambda \left( {\mathcal {C}}_z - \frac{1}{2} ({\mathcal {C}}_\zeta + {\mathcal {C}}_{{\overline{\zeta }}}) \right) \Lambda ; \end{aligned}$$

cf. [9, 19, 22, 23] for details on boundary triples and Weyl functions in the extension theory of symmetric operators.

Next we describe the operators $A_\eta$ with the help of the boundary triple (2.2) and also conclude their self-adjointness in $L^2({\mathbb {R}}^2; {\mathbb {C}}^2)$. The proof of this result follows the lines of [21, Proposition 6.3] and [12, Proposition 4.3].

### Lemma 2.2

For $\eta \in {\mathbb {R}}\setminus \{0\}$ the operator

$$\begin{aligned} \begin{aligned} \Theta \varphi&:= -\Lambda \left[ \frac{1}{\eta }\sigma _0 + \frac{1}{2} ({\mathcal {C}}_\zeta + {\mathcal {C}}_{{\overline{\zeta }}}) \right] \Lambda \varphi , \\ \mathrm {dom}\,\Theta&= \bigg \{ \varphi \in L^2(\Sigma ; {\mathbb {C}}^2): \left[ \frac{1}{\eta }\sigma _0 + \frac{1}{2} ({\mathcal {C}}_\zeta + {\mathcal {C}}_{{\overline{\zeta }}}) \right] \Lambda \varphi \in H^{1/2}(\Sigma ; {\mathbb {C}}^2) \bigg \}, \end{aligned} \end{aligned}$$

is self-adjoint in $L^2(\Sigma ; {\mathbb {C}}^2)$ and we have

2.3 $$\begin{aligned} A_\eta = S^* \upharpoonright \ker (\Gamma _1 - \Theta \Gamma _0). \end{aligned}$$

In particular, the operator $A_\eta$ is self-adjoint in $L^2({\mathbb {R}}^2; {\mathbb {C}}^2)$.

### Proof

Define the ${\mathbb {C}}^{2 \times 2}$-valued function

$$\begin{aligned} \theta (p) := -\sqrt{p^2+1} \begin{pmatrix} \frac{1}{\eta } + Re \, \frac{\zeta + m}{2 \sqrt{p^2+m^2-\zeta ^2}} &{} Re \, \frac{p}{2 \sqrt{p^2+m^2-\zeta ^2}} \\ Re \, \frac{p}{2 \sqrt{p^2+m^2-\zeta ^2}} &{} \frac{1}{\eta } + Re \, \frac{\zeta - m}{2 \sqrt{p^2+m^2-\zeta ^2}} \end{pmatrix}. \end{aligned}$$

Then, by Lemma 2.1 we have

$$\begin{aligned} \begin{aligned} {\mathcal {F}} \Theta {\mathcal {F}}^{-1} \varphi (p)&= \theta (p) \varphi (p), \\ \mathrm {dom}\,{\mathcal {F}} \Theta {\mathcal {F}}^{-1}&= \{ \varphi \in L^2({\mathbb {R}}; {\mathbb {C}}^2): \theta \varphi \in L^2({\mathbb {R}}; {\mathbb {C}}^2) \}. \end{aligned} \end{aligned}$$

Since $\theta$ is a symmetric matrix and ${\mathcal {F}}$ is unitary, we conclude that $\Theta$ is self-adjoint in $L^2(\Sigma ; {\mathbb {C}}^2) \simeq L^2({\mathbb {R}}; {\mathbb {C}}^2)$. Using (2.2) it is not difficult to verify that (2.3) holds and hence the self-adjointness of $A_\eta$ follows, see, e.g., [9, Corollary 2.1.4 (v)].$\square$

In the next lemma we analyze, when zero belongs to the point spectrum or continuous spectrum of $\Theta - M(z)$ for $z \in (-|m|, |m|)$.

### Lemma 2.3

Let $\eta \in {\mathbb {R}}\setminus \{0\}$. For $z \in (-|m|, |m|)$ the following holds:

(i) If $\eta \ne \pm 2$, then $0 \notin \sigma _p (\Theta - M(z))$ and $0 \in \sigma _c (\Theta - M(z))$ if and only if
  2.4 $$\begin{aligned} \frac{z \eta }{\eta ^2-4}>0 \end{aligned}$$
  and
  $$\begin{aligned} z \le - \frac{ |m (\eta ^2-4) |}{\eta ^2+4} \quad \text {or}\quad z \ge \frac{ |m (\eta ^2-4) |}{\eta ^2+4}. \end{aligned}$$
(ii) If $\eta =\pm 2$, then $0 \in \sigma _p (\Theta - M(0))$ with $\dim \ker (\Theta - M(0))=+\infty$, and $0 \in \rho (\Theta - M(z))$ for $z \ne 0$.

### Proof

Let $z \in (-|m|, |m|)$ and observe that

2.5 $$\begin{aligned} \Theta -M(z) = -\Lambda \left[ \frac{1}{\eta }\sigma _0 + {\mathcal {C}}_z \right] \Lambda , \quad \mathrm {dom}\,(\Theta -M(z))=\mathrm {dom}\,\Theta . \end{aligned}$$

Using Lemma 2.1 we conclude

$$\begin{aligned} \begin{aligned} {\mathcal {F}} (\Theta -M(z)) {\mathcal {F}}^{-1} \varphi (p)&= \theta _z(p) \varphi (p), \\ \mathrm {dom}\,{\mathcal {F}} (\Theta -M(z)){\mathcal {F}}^{-1}&= \{ \varphi \in L^2({\mathbb {R}}; {\mathbb {C}}^2): \theta _z \varphi \in L^2({\mathbb {R}}; {\mathbb {C}}^2) \}, \end{aligned} \end{aligned}$$

where

$$\begin{aligned} \theta _{z}(p)=-\sqrt{p^2+1} \begin{pmatrix} \frac{1}{\eta } + \frac{z + m}{2 \sqrt{p^2+m^2-z^2}} &{} \frac{p}{2 \sqrt{p^2+m^2-z^2}} \\ \frac{p}{2 \sqrt{p^2+m^2-z^2}} &{} \frac{1}{\eta } + \frac{z - m}{2 \sqrt{p^2+m^2-z^2}} \end{pmatrix}, \end{aligned}$$

and hence it suffices to consider the self-adjoint multiplication operator with the function $\theta _z$ in $L^2({\mathbb {R}}; {\mathbb {C}}^2)$. In the following we discuss for which $\eta \in {\mathbb {R}}\setminus \{0\}$ and $z\in (-|m|, |m|)$ the multiplication operator $\theta _z$ has 0 as an eigenvalue or as a point in the continuous spectrum. Note first that

$$\begin{aligned} \det \theta _z(p) = (p^2+1) \left[ \frac{1}{\eta ^2}+ \frac{z}{\eta \sqrt{p^2+m^2-z^2}}-\frac{1}{4}\right] . \end{aligned}$$

We verify assertion (ii). In the case $\eta =\pm 2$ we have $\det \theta _z(p)=0$ if and only if $z=0$, and in this situation $\det \theta _z(p)=0$ for all $p\in {\mathbb {R}}$. Therefore, 0 is an eigenvalue of infinite multiplicity of the multiplication operator $\theta _z$. Observe that for $z \in (-|m|, |m|)\setminus \{0\}$ we have $\det \theta _z(p)\not =0$ and hence one finds that 0 is in the resolvent set of $\theta _z$.

Now we prove (i). If $\eta \ne \pm 2$, then $\det \theta _z(p)=0$ is equivalent to

2.6 $$\begin{aligned} \begin{aligned} \sqrt{p^2+m^2-z^2}=4\frac{z\eta }{\eta ^2-4}. \end{aligned} \end{aligned}$$

The left hand side of the last equation is positive and hence there exist solutions only if also the right hand side is positive, i.e. only if (2.4) holds. Assuming this, we see by squaring the last equation that it is equivalent to

2.7 $$\begin{aligned} p^2 = \frac{(\eta ^2 + 4)^2}{(\eta ^2 - 4)^2}\,z^2 - m^2. \end{aligned}$$

When $p \in {\mathbb {R}}$ varies the left hand side can be any non-negative number, and hence (2.6) has a solution, whenever the right hand side is non-negative, i.e. if and only if $z \le z_-$ or $z \ge z_+$, where $z_{\pm }$ are the zeros of the polynomial on the right hand side of the last equation given by

$$\begin{aligned} z_{\pm } = \pm \left| m \frac{\eta ^2 -4}{\eta ^2 +4}\right| . \end{aligned}$$

It is also clear that for a fixed $z \in (-|m|, |m|)$ with $z \le z_-$ or $z \ge z_+$ there are only two values $p\in {\mathbb {R}}$ with (2.7) holds, and hence 0 is in the continuous spectrum of the multiplication operator associated with $\theta _z$.$\square$

In order to prove Theorem 1.1, we also investigate the limiting behaviour of $( \Theta - M(z) )^{-1}$, when $z \in {\mathbb {C}} \setminus {\mathbb {R}}$ approaches $\sigma (A_0) = (-\infty , -|m|] \cup [|m|, +\infty )$:

### Lemma 2.4

Let $\eta \in {\mathbb {R}} \setminus \{ 0 \}$. Then, the following is true:

(i) For any $x \in (-\infty , -|m|] \cup [|m|, +\infty )$ and all $\varphi \in L^2(\Sigma ; {\mathbb {C}}^2)$ one has
  2.8 $$\begin{aligned} \lim _{y \searrow 0} i y \big ( \Theta - M(x+iy) \big )^{-1} \varphi = 0. \end{aligned}$$
(ii) For any $x \in (-\infty , -|m|) \cup (|m|, +\infty )$ there exists $\varphi \in L^2(\Sigma ; {\mathbb {C}}^2)$ such that
  2.9 $$\begin{aligned} \lim _{y \searrow 0} Im \, \big ( \big ( \Theta - M(x+iy) \big )^{-1} \varphi , \varphi \big ) \ne 0. \end{aligned}$$

### Proof

In order to show the claims, we note first with the help of (2.5) that the operator ${\mathcal {F}} (\Theta -M(z))^{-1} {\mathcal {F}}^{-1}$, $z \in {\mathbb {C}} \setminus {\mathbb {R}}$, is the maximal multiplication operator associated with the matrix-valued function

$$\begin{aligned} \begin{aligned}&\theta _{z}^{-1}(p)= -\frac{\sqrt{p^2+1}}{\det \theta _z(p)} \begin{pmatrix} \frac{1}{\eta } + \frac{z - m}{2 \sqrt{p^2+m^2-z^2}} &{} -\frac{p}{2 \sqrt{p^2+m^2-z^2}} \\ -\frac{p}{2 \sqrt{p^2+m^2-z^2}} &{} \frac{1}{\eta } + \frac{z + m}{2 \sqrt{p^2+m^2-z^2}} \end{pmatrix} \\&~~={ -\frac{2 \eta }{c_z(p) \sqrt{p^2\!+\!1}} \! \begin{pmatrix} 2 \sqrt{p^2\!+\!m^2\!-\!z^2} \!+\! \eta (z\! -\! m) &{} -\eta p \\ -\eta p &{} 2 \sqrt{p^2\!+\!m^2\!-\!z^2} \!+\! \eta (z \!+\! m) \end{pmatrix} } \end{aligned} \end{aligned}$$

with

$$\begin{aligned} c_z(p) = (4-\eta ^2)\sqrt{p^2+m^2-z^2} + 4\eta z. \end{aligned}$$

By the continuity of the complex square root one sees for a fixed $p \in {\mathbb {R}}$ that the limit $c_{x+i0}(p) := \lim _{y \searrow 0} c_{x+iy}(p)$ exists. One verifies for any number $x \in (-\infty , -|m|] \cup [|m|, +\infty )$ in a similar way as in (2.6) and (2.7) that $c_{x+i0}(p)$ has no zero, if $\eta = \pm 2$ or if $\frac{x \eta }{\eta ^2-4}<0$, and for $\frac{x \eta }{\eta ^2-4}>0$ the term $c_{x+i0}(p)$ has two zeros at

2.10 $$\begin{aligned} p_\pm = \pm \left( \frac{(\eta ^2 + 4)^2}{(\eta ^2 - 4)^2}\,x^2 - m^2 \right) ^{1/2}. \end{aligned}$$

Let us prove (i). Let $x \in (-\infty , -|m|] \cup [|m|, +\infty )$ be fixed. Since the map $z\mapsto M(z)$ is the Weyl function of a boundary triple it is a Nevanlinna function and the values *M*(*z*) are bounded and everywhere defined operators in $L^2(\Sigma ; {\mathbb {C}}^2)$; cf. [9, Corollary 2.3.7]. It follows that $z\mapsto (\Theta -M(z))^{-1}$ is also a Nevanlinna function and for $z\in \rho (A_\eta )\cap \rho (A_0)$ the values are also bounded and everywhere defined operators in $L^2(\Sigma ; {\mathbb {C}}^2)$. From the operator representation of Nevanlinna functions (see, e.g., [25, Theorem 4.2]) we then conclude that there exists a constant $C_1 > 0$ such that

$$\begin{aligned} \big \Vert (\Theta -M(x+iy))^{-1} \big \Vert \le \frac{C_1}{y} \end{aligned}$$

for $y>0$ sufficiently small. Since $(\Theta -M(x+iy))^{-1}$ is unitarily equivalent to the multiplication operator with the function $\theta _{x+iy}^{-1}$, we conclude that

2.11 $$\begin{aligned} \big \Vert \theta _{x+iy}^{-1} \big \Vert _\infty \le \frac{C_1}{y} \end{aligned}$$

for all $y>0$ sufficiently small. Next,we define for $y>0$ the set

2.12 $$\begin{aligned} I_x(y) := {\left\{ \begin{array}{ll} \{ p \in {\mathbb {R}}: |p-p_\pm | \ge \sqrt{y} \}, &{} \text {if } c_{x+i0} \text { has a zero}, \\ {\mathbb {R}}, &{} \text {if } c_{x+i0} \text { has no zero}, \end{array}\right. } \end{aligned}$$

and prove for all sufficiently small $y>0$ and $p \in I_x(y)$ that

2.13 $$\begin{aligned} \big | \theta _{x+iy}^{-1}(p) \big | \le \frac{C_2}{\sqrt{y}} \end{aligned}$$

for some $C_2 > 0$, where the absolute value is understood elementwise.

In order to show (2.13), we note first that for some $C_3>0$ independent of $p \in {\mathbb {R}}$ and $y \in [0,1]$ one has the estimate

2.14 $$\begin{aligned} \big | c_{x+iy}(p) \theta _{x+iy}^{-1}(p) \big | \le C_3. \end{aligned}$$

Consider first the case when $c_{x+i0}$ has no zero. From the definition of $c_{x+iy}$ we conclude that there exists $P_0 > 0$ such that $\big | c_{x+iy}(p) \big | > 1$ holds for all $|p| > P_0$ and all sufficiently small $y>0$. Moreover, the map $[-P_0, P_0] \times [0, 1] \ni (p,y) \mapsto c_{x+iy}(p)$ is uniformly continuous and hence, there exists a constant $C_4 > 0$ such that for all sufficiently small $y > 0$ and $p \in [-P_0, P_0]$ the relation $|c_{x+iy}(p)| > C_4$ holds, as $c_{x+i0}$ has no zero. Thus, for $y>0$ sufficiently small $|c_{x+iy}|$ is uniformly bounded from below by a positive constant, which together with (2.14) leads to the estimate in (2.13).

Assume now that $c_{x+i0}$ has the zeros $p_\pm$, let $y>0$ be sufficiently small, and fix $p \in {\mathbb {R}}$ with $|p-p_\pm | \ge \sqrt{y}$. From (2.10) we get $\sqrt{p_\pm ^2 + m^2 - x^2} > 0$ and thus,

$$\begin{aligned} \begin{aligned}&\bigg |\sqrt{p^2+m^2-(x+iy)^2} - \sqrt{p_\pm ^2+m^2-x^2}\bigg | \\&= \left| \frac{p^2-(x+iy)^2 - p_\pm ^2 + x^2}{\sqrt{p^2+m^2-(x+iy)^2} + \sqrt{p_\pm ^2+m^2-x^2}} \right| \\ {}&\ge \left| \frac{(p-p_\pm )(p + p_\pm )}{\sqrt{p^2+m^2-(x+iy)^2} + \sqrt{p_\pm ^2+m^2-x^2}} \right| - \left| \frac{x^2-(x+iy)^2}{\sqrt{p_\pm ^2+m^2-x^2}} \right| \\ {}&\ge C_5 \sqrt{y} \end{aligned} \end{aligned}$$

with a constant $C_5>0$, where we used in the first inequality for the denominator that the real part of the complex square root is positive and in the last estimate that the functions

$$\begin{aligned} {\mathbb {R}}_{\pm } \ni p \mapsto \left| \frac{p \pm |p_\pm |}{\sqrt{p^2+m^2-(x+iy)^2} + \sqrt{p_\pm ^2+m^2-x^2}} \right| \end{aligned}$$

are uniformly bounded from below independently of $y>0$ sufficiently small. Hence, we conclude for those $y>0$ that

$$\begin{aligned} \big | c_{x+iy}(p)\big | = \big | c_{x+iy}(p)-c_{x+i0}(p_\pm ) \big | \ge C_6 \sqrt{y}. \end{aligned}$$

Together with (2.14) this yields (2.13).

Now, we have everything in hands to prove item (i). Let $\chi$ be the characteristic function for the set $I_x(y)$ defined in (2.12). Then (2.11) and (2.13) imply for an arbitrary $\varphi \in L^2(\Sigma ; {\mathbb {C}}^2)$

$$\begin{aligned} \begin{aligned} \lim _{y \searrow 0}&\big \Vert y\theta _{x+iy}^{-1} \varphi \big \Vert _{L^2({\mathbb {R}}; {\mathbb {C}}^2)} \le \lim _{y \searrow 0} \big \Vert y (1-\chi ) \theta _{x+iy}^{-1} \varphi \big \Vert _{L^2({\mathbb {R}}; {\mathbb {C}}^2)} + \lim _{y \searrow 0} \big \Vert y \chi \theta _{x+iy}^{-1} \varphi \big \Vert _{L^2({\mathbb {R}}; {\mathbb {C}}^2)} \\&\le \lim _{y \searrow 0} C_1 \big \Vert (1-\chi ) \varphi \big \Vert _{L^2({\mathbb {R}}; {\mathbb {C}}^2)} + \lim _{y \searrow 0} C_2 \sqrt{y} \big \Vert \varphi \big \Vert _{L^2({\mathbb {R}}; {\mathbb {C}}^2)} =0. \end{aligned} \end{aligned}$$

Thus, assertion (i) is true.

To show item (ii), let $x \in (-\infty , -|m|) \cup (|m|, +\infty )$ and let *I* be a non-empty compact interval which is contained in $(-\sqrt{x^2-m^2},\sqrt{x^2-m^2})$. Note that the numbers $p_\pm$ in (2.10) are not contained in *I* and that

$$\begin{aligned} \lim _{y \searrow 0} \text {Im}\, \sqrt{p^2 + m^2 - (x+iy)^2} \ne 0 \end{aligned}$$

for $p \in I$. Let ${\widetilde{\chi }}$ be the characteristic function for *I*. Taking the form of $\theta _{x+iy}^{-1}$ into account, we get that $\text {Im}\, ({\widetilde{\chi }} \theta _{x+iy}^{-1})$ converges uniformly to a nontrivial limit and hence, one can choose $\varphi \in L^2(\Sigma ; {\mathbb {C}}^2)$ supported in *I* such that

$$\begin{aligned} \lim _{y \searrow 0} Im \, \big ( \big ( \Theta - M(x+iy) \big )^{-1} \varphi , \varphi \big ) \ne 0, \end{aligned}$$

i.e. also item (ii) is true.$\square$

### Proof

(Proof of Theorem 1.1) First, we note that the case $\eta =0$ reduces to the free Dirac operator and hence, all claims are known here. So we assume from now on that $\eta \ne 0$. The self-adjointness of $A_\eta$ is shown in Lemma 2.2. The claims about $\sigma (A_\eta ) \cap (-|m|, |m|)$ follow from Lemma 2.3 and [9, Theorem 2.6.2].

It remains to show for $\eta \in {\mathbb {R}} \setminus \{ 0 \}$ that $(-\infty , -|m|] \cup [|m|, +\infty )$ is purely continuous spectrum of $A_\eta$. For this purpose let *S* be the operator from (2.1) and define the operator $T := S^* \upharpoonright (\mathrm {dom}\,A_\eta + \mathrm {dom}\,A_0)$. Let $\Gamma _0, \Gamma _1$ be the mappings from (2.2) and define $\Gamma _0^\Theta , \Gamma _1^\Theta : \mathrm {dom}\,T \rightarrow L^2(\Sigma ; {\mathbb {C}}^2)$ by

$$\begin{aligned} \Gamma _0^\Theta f := \Gamma _1 f - \Theta \Gamma _0 f, \quad \Gamma _1^\Theta f := -\Gamma _0 f, \quad f \in \mathrm {dom}\,T. \end{aligned}$$

Similarly as in [11, Proposition 2.4] one verifies that $\{L^2(\Sigma ; {\mathbb {C}}^2), \Gamma _0^\Theta , \Gamma _1^\Theta \}$ is a quasi boundary triple for $S^*$ in the sense of [14] with $T \upharpoonright \ker \Gamma _0^\Theta = A_\eta$ and Weyl function

$$\begin{aligned} \rho (A_\eta ) \ni z \mapsto -\big (\Theta - M(z)\big )^{-1}. \end{aligned}$$

Note that the operator *S* in (2.1) is simple; this can be shown as in [11, Proposition 3.2] using [16, Step 2 in the proof of Theorem 3.4]. Hence, Lemma 2.4 (i) and [15, Theorem 3.2] imply that $(-\infty , -|m|] \cup [|m|, +\infty ) \cap \sigma _\text {p}(A_\eta )=\emptyset$. Furthermore, Lemma 2.4 (ii) and [15, Theorem 3.5] yield that the inclusion $(-\infty , -|m|) \cup (|m|, +\infty ) \subset \sigma _\text {c}(A_\eta )$ holds. By combining these facts one shows that $(-\infty , -|m|] \cup [|m|, +\infty )$ is contained in the purely continuous spectrum of $A_\eta$. $\square$

## Data Availability

Data sharing not applicable to this article as no datasets were generated or analysed during the current study.
